# Machine learning methods in the computational biology of cancer

**DOI:** 10.1098/rspa.2014.0081

**Published:** 2014-07-08

**Authors:** M. Vidyasagar

**Affiliations:** Erik Jonsson School of Engineering and Computer Sciences, University of Texas at Dallas, 800 West Campbell Road, Richardson, TX 75080, USA

**Keywords:** cancer biology, machine learning, support vector machines, LASSO algorithm, elastic net, algorithm, compressed sensing

## Abstract

The objectives of this Perspective paper are to review some recent advances in sparse feature selection for regression and classification, as well as compressed sensing, and to discuss how these might be used to develop tools to advance personalized cancer therapy. As an illustration of the possibilities, a new algorithm for sparse regression is presented and is applied to predict the time to tumour recurrence in ovarian cancer. A new algorithm for sparse feature selection in classification problems is presented, and its validation in endometrial cancer is briefly discussed. Some open problems are also presented.

## 1. Introduction

The objectives of this Perspective paper are to review some recent advances in sparse feature selection for regression and classification, and to discuss how these might be used in the computational biology of cancer. One of the motivations for writing this paper is to present a broad picture of some recent advances in machine learning to the more mathematically inclined within the cancer biologist community, and to apply some of these techniques to a couple of problems. Full expositions of these applications will be presented elsewhere. In the other direction, it is hoped that the paper will also facilitate the entry of interested researchers from the machine learning community into cancer biology. In order to understand the *computational* aspects of the problems described here, a basic grasp of molecular biology is sufficient, as can be obtained from standard references, for example Northrop & Connor [1] and Tözeren & Byers [2].

Cancer is the second leading cause of death in the USA [3]. It is estimated that in the USA in 2013 there will be 1 660 290 new cases of cancer in all sites, and 589 350 deaths [4]. In the UK, in 2011 there were 331 487 cases of cancer, and 159 178 deaths; both are the latest figures available [5]. Worldwide, cancer led to about 7.6 million deaths in 2008 [6]. It is interesting to note that, whether in developed countries such as the USA and the UK or worldwide, cancer accounts for roughly 13% of all deaths [6].

One of the major challenges faced by cancer researchers is that no two manifestations of cancer are alike, even when they occur in the same site. One can paraphrase the opening sentence of Leo Tolstoy's *Anna Karenina* and say that ‘Normal cells are all alike. Every malignant cell is malignant in its own way.’ Thus, cancer would be an ideal target for ‘personalized medicine’, in which therapy is custom-tailored to each patient. Unfortunately, our current level of understanding of the disease does not permit us to develop truly personalized therapies for every individual patient. Therefore, it is necessary to settle for an intermediate approach, which might be described as ‘patient stratification’. In this approach, diverse manifestations of a particular type of cancer are grouped into a small number of classes, wherein the manifestations are broadly similar within each class and substantially different between classes. Then attempts can be made to develop therapeutic regimens that are tailored for each class.

Until recently, grouping of cancers has been attempted first through the site of the cancer, and then through histological considerations, that is, the microscopic anatomy of the cells comprising the tumour, and other parameters that can be measured by a physical examination of the tumour. For example, lung cancer is divided into two broad categories, namely small-cell lung cancer (SCLC) and non-small-cell lung cancer (NSCLC), where the prognosis for the latter is decidedly better than for the former. Then, NSCLC is divided into three subtypes known as adenocarcinoma, squamous cell carcinoma and large-cell carcinoma. All of these subtypes are defined on the basis of histology. But this is not the only possible approach. It is also possible to define the subtypes on the basis of the molecular-level properties of the cancer tumour. For instance, there are four major types of breast cancer, known as luminal A, luminal B, non-luminal and basal type. These subtypes are defined based on the expression levels of the genes oestrogen receptor, progesterone receptor and HER2, also known as ERBB2, being either high or low. The basal-like subtype, also known as the triple negative subtype owing to the fact that all three genes are expressed at very low levels, constitutes about 20% of breast cancer cases and has the worst prognosis. For the other three subtypes, there are some proved therapies that work reasonably well; but this is not so for triple negative subtypes. The above subtyping illustrates the type of challenges faced by a mathematically trained person when studying computational biology. For instance, given that there are three genes being studied, and that the expression level of each can be either high or low, a mathematician/engineer might think that there are 2^3^=8 possible subtypes. In reality however, as stated above, there are only four subtypes, and some of the possible combinations do not seem to occur sufficiently frequently.^1^ The therapies for the various subtypes are quite different. Therefore, it is important to be able to ascertain to which subtype a patient belongs, before commencing therapy. This is one possible application of machine learning.

During the past decade, attempts have been made to collect the experimental data generated by various research laboratories into central repositories such as the Gene Expression Omnibus [8] and the Catalogue of Somatic Mutations in Cancer (COSMIC) [9]. However, the data in these repositories are often collected under widely varying experimental conditions. Moreover, the standard of reporting and documentation is not always uniform. To mitigate this problem, there are now some massive public projects underway for generating vast amounts of data for all the tumours that are available in various tumour banks, using standardized sets of experimental protocols. Among the most ambitious are The Cancer Genome Atlas, usually referred to by the acronym TCGA [10], which is undertaken by the National Cancer Institute, and the International Cancer Genome Consortium, referred to also as ICGC [11], which is a multi-country effort. In the TCGA data, molecular measurements are available for almost all tumours, and clinical annotations are also available for many tumours.

With such a wealth of data becoming freely available, researchers in the machine learning community can now aspire to make useful contributions to cancer biology without the need to undertake any experimentation themselves. However, in order to carry out meaningful research, it is essential to have a close collaboration with one or more biologists. The style of exposition in the biological literature is quite different from that in mathematical books and papers, and the author's experience has been that simply conversing with expert biologists is the fastest way to become familiar with the subject.^2^ Unlike in mathematics, in biology it is *not* possible to derive everything from a few fundamental axioms and/or principles; instead, one is confronted with a bewildering variety of terms and names, all of which have to be mastered (memorized?) in parallel. One example, as mentioned above in connection with breast cancer, is that the names ERBB2, HER2 and HER2/Neu all refer to the same gene. Also, while it is not necessary to perform experiments oneself, it is absolutely crucial to understand *the nature of the experiments*, so that one is aware of the potential sources of error and the level of reliability of specific types of molecular measurements.

Owing to space limitations, in this paper only two out of the many possible applications of machine learning to cancer are addressed, namely sparse regression and sparse classification. Other topics such as network inference and modelling tumour growth are mentioned very briefly in passing towards the end of the paper.

Now, we briefly state the class of problems under discussion in this paper. This also serves to define the notation used throughout. Let *m* denote the number of tumour samples that are analysed, and let *n* denote the number of attributes, referred to as ‘features’, that are measured on each sample. Typically, *m* is of the order of a few dozen in small studies, ranging up to several hundreds for large studies such as the TCGA studies, while *n* is of the order of tens of thousands. There are 20 000 or so genes in the human body, and in whole genome studies, and the expression level of each gene is measured by at least one ‘probe’, and sometimes by more than one. The ‘raw’ expression level of a gene corresponds to the amount of messenger RNA that is produced and is therefore a non-negative number. However, the raw value is often transformed by taking the logarithm after dividing by a reference value, subtracting a median value, dividing by a scaling constant and the like. As a result, the numbers that are reported as gene expression levels can sometimes be negative numbers. Therefore, it is best to think of gene expression levels as real numbers. Other features that are measured include micro-RNA (miRNA) levels, methylation levels and copy number variations, all of which can be thought of as real-valued. There are also binary features such as the presence or absence of a mutation in a specific gene. In addition to these molecular attributes, there are also ‘labels’ associated with each tumour. Let *y*_*i*_ denote the label of tumour *i*, and note that the label depends only on the sample index *i* and not the feature index *j*. Typical real-valued labels include the time of overall survival after surgery, time to tumour recurrence or the lethality of a drug on a cancer cell line. Typical binary labels include whether a patient had metastasis (cancer spreading beyond the original site). In addition, it is also possible for labels to be ordinal variables, such as ‘poor responder’, ‘medium responder’ and ‘good responder’. Often these ordinal labels are merely quantized versions of some other real-valued attributes. For instance, the previous example corresponds to a three-level quantization of the time to tumour recurrence. In general, the labels refer to *clinical outcomes*, as in all of the above examples. Usually, each sample has multiple labels associated with it. However, in applications, the labels are treated one at a time, so it is assumed that there is only one label for each sample, with *y*_*i*_ denoting the label of the *i*th sample. Moreover, for simplicity, it is assumed that the labels are either real-valued or binary.

Thus, the measurement set can be thought of as an *m*×*n* matrix *X*=[*x*_*ij*_], where *x*_*ij*_ is the value of feature *j* in sample *i*. The row vector *x*^*i*^, denoting the *i*th row of the matrix *X*, is called the feature vector associated with sample *i*. Similarly, the column vector *x*_*j*_ denotes the variation of the *j*th feature across all *m* samples. Throughout this paper, it is assumed that $X\in R^{m\times n}$, that is, that each measurement is a real number. Binary measurements such as the presence or absence of mutations are usually handled by partitioning the sample set into two groups, corresponding to the two labels. For the purposes of incorporating binary labels into numerical computation, the labels are taken as ±1, the so-called bipolar case. It does not matter which abstract label is mapped into +1 and which abstract label is mapped into −1. If *y*_*i*_ is bipolar, the associated problem is called ‘classification’, whereas if *y*_*i*_ is real the associated problem is called ‘regression’. In either case, the objective is to find a function $f:R^{n}\to R$ or $f:R^{n}\to \{-1,1\}$ such that *y*_*i*_ is well approximated by *f*(*x*^*i*^).

## 2. Regression methods

The focus in this section is on the case where the label *y*_*i*_ is a real number. Therefore, the objective is to find a function $f:R^{n}\to R$ such that *f*(*x*^*i*^) is a good approximation of *y*_*i*_ for all *i*. A typical application in cancer biology would be the prediction of the time for a tumour to recur after surgery. The data would consist of expression levels of tens of thousands of genes on around a hundred or so tumours, together with the time for the tumour to recur for each patient. The objective is to identify a small number of genes whose expression values would lead to a reliable prediction of the recurrence time. Cancer is a complex, multi-genic disease, and identifying a small set of genes that appear to be highly predictive in a particular form of cancer would be very useful. Explaining *why* these genes are the key genes would require constructing gene regulatory networks (GRNs). While this problem is also amenable to treatment using statistical methods, it is beyond the scope of this paper. Towards the end of this section, the tumour recurrence problem is studied using a new regression method.

### (a) Some well-established algorithms

Throughout this section, attention is focused on *linear regressors*, with *f*(*x*)=*xw*−*θ*, where $w\in R^{n}$ is a weight vector and θ∈R is a threshold or bias. There are several reasons for restricting attention to linear regressors. From a mathematical standpoint, linear regressors are by far the most widely studied and the best understood class of regressors. From a biological standpoint, it makes sense to suppose that the measured outcome is a weighted linear combination of each feature, with perhaps some offset term. If one were to use higher order polynomials, for example, then biologists would rightly object that taking the product of two features (say two gene expression values) is unrealistic most of the time.^3^ Other possibilities include pre-processing each feature *x*_*ij*_ through a function such as *x*↦e^*x*^/(1+e^*x*^), but this is still linear regression in terms of the processed values. As explained earlier, often the measured feature values *x*_*ij*_ are themselves processed values of the corresponding ‘raw’ measurements.

In traditional least-squares regression, the objective is to choose a weight vector $w\in R^{n}$ and a threshold *θ* so as to minimize the least-squared error

2.1 $$J_{\mathrm{LS}}:=\sum_{i=1}^{m}{\left(x^{i}w-\theta -y_{i}\right)}^{2}.$$

This method goes back to Legendre and Gauss and is the staple of researchers everywhere. Let ***e*** denote a column vector of all ones, with the subscript denoting the dimension. Then,

$$J_{\mathrm{LS}}=∥Xw-\theta e_{m}-y{∥}_{2}^{2}=∥\bar{X}\bar{w}-y{∥}_{2}^{2},$$

where

$$\bar{X}=[X\;-e_{m}]\in R^{m\times (n+1)}\;\mathrm{and}\;\bar{w}=\left[\begin{array}{c} w\\ \theta \end{array}\right]\in R^{n+1}.$$

If the matrix $\bar{X}$ has full column rank of *n*+1, then it is easy to see that the unique optimal choice ${\bar{w}}^{*}$ is given by

$${\bar{w}}_{\mathrm{LS}}^{*}={\left({\bar{X}}^{t}\bar{X}\right)}^{-1}{\bar{X}}^{t}y={\left[\begin{array}{cc} X^{t}X & -X^{t}e_{m}\\ -e_{m}^{t}X & m \end{array}\right]}^{-1}\left[\begin{array}{c} X^{t}\\ e_{m}^{t} \end{array}\right]y.$$

In the present context, the fact that *m*<*n* ensures that the matrix *X* has rank less than *n*, whence the matrix $\bar{X}$ has rank less than *n*+1. As a result, the standard least-squares regression problem does not have a unique solution. Therefore, one attempts to minimize the least-squares error while imposing various constraints (or penalties) on the weight vector *w*.^4^ Different constraints lead to different problem formulations. An excellent and very detailed treatment of the various topics of this section can be found in Hastie *et al.* [12, ch. 3].

Suppose we minimize the least-squared error objective function subject to an ℓ_2_-norm constraint on *w*. This approach to finding a unique set of weights is known as ‘ridge regression’ and is usually credited to Hoerl & Kennard [13]. However, several of the key ideas are found in a much earlier paper by the Russian mathematician Tikhonov [14]. In ridge regression, the problem is reformulated as

$$\min\sum_{i=1}^{m}{\left(x^{i}w-\theta -y_{i}\right)}^{2}\;\text{ s.t. }∥w{∥}_{2}\leq t,$$

where *t* is some prespecified bound. In the associated Lagrangian formulation, the problem becomes one of the minimizing objective function

2.2 $$J_{\mathrm{ridge}}:=\sum_{i=1}^{m}{\left(x^{i}w-\theta -y_{i}\right)}^{2}+\lambda ∥w{∥}_{2}^{2},$$

where λ is the Lagrange multiplier. Because of the additional term, the (1,1)-block of the Hessian of *J*_*ridge*_, which is the Hessian of *J*_*ridge*_ with respect to *w*, now equals λ*I*_*n*_+*X*^*t*^*X*, which is positive definite even when *m*<*n*. Therefore, the overall Hessian matrix is positive definite under a mild technical condition, and the problem has a unique solution for every value of the Lagrange parameter λ. However, the major disadvantage of ridge regression is that, in general, *every component* of the optimal weight vector *w*_*ridge*_ is non-zero. In the context of biological applications, this means that the regression function makes use of *every* feature *x*_*j*_, which is in general undesirable.

Another possibility is to choose a solution *w* that has the fewest number of non-zero components, that is, a regressor that uses the fewest number of features. Define

$$∥w{∥}_{p}:={\left(\sum_{i=1}^{n}|w_{i}{|}^{p}\right)}^{1/p}.$$

If *p*≥1, this is the familiar ℓ_*p*_-norm. If *p*<1, this quantity is no longer a norm, as the function *w*↦∥*w*∥_*p*_ is no longer convex. However, as *p*↓0, the quantity ∥*w*∥_*p*_ approaches the number of non-zero components of *w*. For this reason, it is common to refer to the number of non-zero components of a vector as its ‘ℓ_0_-norm’ even though ∥⋅∥_0_ is not a norm at all. Moreover, it is known [15] that the problem of minimizing ∥*w*∥_0_ is NP-hard.

A very general formulation of the regression problem is to minimize

2.3 $$J_{M}:=\sum_{i=1}^{m}{\left(x^{i}w-\theta -y_{i}\right)}^{2}+R(w),$$

where $R:R^{n}\to R_{+}$ is a norm known as the ‘regularizer’. This problem is analysed at a very high level of generality in Negabhan *et al.* [16], where the least-squares error term is replaced by an arbitrary convex ‘loss’ function. In the interests of simplicity, we do not discuss the results of Negabhan *et al.* [16] in their full generality and restrict the discussion to the case where the loss function is quadratic as in (2.3).

In Tibshirani [17], it is proposed to minimize the least-squared error objective function subject to an ℓ_1_-norm constraint on the weight vector *w*. In Lagrangian formulation, the problem is to minimize

2.4 $$J_{\mathrm{LASSO}}:=\sum_{i=1}^{m}{\left(x^{i}w-\theta -y_{i}\right)}^{2}+\lambda ∥w{∥}_{1},$$

where λ is the Lagrange multiplier. The acronym ‘LASSO’ is coined in Tibshirani [17] and stands for ‘least absolute shrinkage and selection operator’. The LASSO penalty can be rationalized by observing that ∥⋅∥_1_ is the convex relaxation of the ‘ℓ_0_-norm’. The behaviour of the solution to the LASSO algorithm depends on the choice of the upper bound *t*. A detailed analysis of the Lagrangian formulation (2.4) and its dual problem is carried out in Osborne *et al.* [18]. It is shown there that, if the Lagrange multiplier λ in (2.4) is sufficiently large, say λ>λ_*max*_, then the only solution to the LASSO minimization problem is *w*=0. Moreover, the threshold λ_*max*_ is not easy to estimate *a priori*. An optimal solution is defined to be ‘regular’ in Osborne *et al.* [18, definition 3.3] if it satisfies some technical conditions. In every problem, there is at least one regular solution. Moreover, every regular optimal weight vector has at most *m* non-zero entries (see Osborne *et al.* [18, theorem 3.5]).

In many applications, some of the columns of the matrix *X* are highly correlated. For instance, if the indices *j* and *k* correspond to two genes that are in the same biological pathway, then their expression levels would vary in tandem across all samples. Therefore, the column vectors *x*_*j*_ and *x*_*k*_ would be highly correlated. In such a case, ridge regression tends to assign nearly equal weights to each. At the other extreme, LASSO tends to choose just one among the many correlated columns and to discard the rest; which one gets chosen is often a function of the ‘noise’ in the measurements. In biological datasets, it is reasonable to expect that expression levels of genes that are in a common pathway are highly correlated. In such a situation, it is undesirable to choose just one among these genes and to discard the rest; it is also undesirable to choose all of them, as that would lead to too many features being chosen. It would be desirable to choose more than one, but not all, of the correlated columns. This is achieved by the so-called ‘elastic net’ (EN) algorithm, introduced in Zou & Hastie [19], which is a variation of the LASSO algorithm. In this algorithm, the penalty aims to constrain, not the ℓ_1_-norm of the weight *w*, but a weighted sum of its ℓ_1_-norm and ℓ_2_-norm squared. The problem formulation in this case, in Lagrangian form, is to choose *w* so as to minimize

2.5 $$J_{\mathrm{EN}}:=\sum_{i=1}^{n}{\left(x^{i}w-\theta -y_{i}\right)}^{2}+\lambda [\mu ∥w{∥}_{2}^{2}+(1-\mu )∥w{∥}_{1}],$$

where *μ*∈(0,1). Note that if *μ*=0, then the EN algorithm becomes the LASSO, whereas with *μ*=1, the EN algorithm becomes ridge regression. Thus, the EN algorithm provides a bridge between the two. Note that the penalty term in the EN algorithm is *not* a norm, owing to the presence of the squared term; hence, the EN algorithm is not covered by the very thorough analysis in Negabhan *et al.* [16]. A useful property of the EN algorithm is brought out in Zou & Hastie [19, theorem 1].

**Theorem 2.1**

*Assume that y,X*,λ *are fixed, and let* $\bar{w}$ *denote the corresponding minimizer of* (2.5). *Assume without loss of generality that y is centred, that is, y*^*t*^***e***_*m*_*=0, and that the columns of X are normalized such that ∥x*_*j*_*∥*_2_*=1 for all j. Let j,k be two indices between 1 and n, and suppose that* $x_{j}^{t}x_{k}\geq 0$*. Then,*

2.6 $$|w_{j}-w_{k}|\leq \frac{∥y{∥}_{1}}{\lambda \mu }\sqrt{2(1-x_{j}^{t}x_{k})}.$$

As one can always ensure that $x_{j}^{t}x_{k}^{t}\geq 0$ by replacing *x*_*k*_ by −*x*_*k*_ if necessary, (2.6) states that if the columns *x*_*j*_ and *x*_*k*_ are highly correlated, then the corresponding coefficients in the regressor are nearly equal. Unlike in the LASSO algorithm, there do not seem to be many results on the number of non-zero weights that are chosen by the EN algorithm. It can and often does happen that the number of features chosen is larger than *m*, the number of samples. However, as explained above, this is often seen as a desirable feature when the columns of the matrix *X* are highly correlated, as they often are in biology datasets.

By now both LASSO and EN can be viewed as well-established algorithms. A search of the Pubmed database of the National Library of Medicine with strings ‘LASSO cancer’ or ‘EN cancer’ results in about 200 entries for the former and several dozen entries for the latter. Note that these numbers are an order of magnitude less than the corresponding numbers for the support vector machine (SVM), discussed in §2*b*. Many of the papers citing the LASSO algorithm do not directly apply the algorithm to cancer data; instead, they propose some variant of the algorithm and claim to show that their variant outperforms the standard LASSO algorithm. A surprisingly large number of these variants propose non-convex objective functions (such as the ‘ℓ_*p*_-norm’ with *p*<1). Given that, in convex optimization, every local optimum is also a global optimum, whereas this is not so in the case of non-convex optimization, it is difficult to imagine what benefits if any are conferred by replacing the convex objective function *J*_LASSO_ with a non-convex objective function. But there are many such papers to be found in the literature. In the case of the EN algorithm, a typical application is found in Lee *et al.* [20] that addresses the problem of identifying some genes to delineate advanced versus early stage colorectal cancer. In this study, 1192 known or putative cancer genes found from Network [21] and COSMIC [9] constitute the feature set on 197 samples. As expected, the EN algorithm chooses a large number of features, which are then rank-ordered to determine the key genes. An interesting paper [22] compares all the three methods discussed here, namely ridge regression, LASSO and EN, on several datasets both synthetic and real, including a lung adenocarcinoma dataset. Not surprisingly, ridge regression assigns a non-zero weight to all 1310 features, whereas EN assigns zero weights to only 43 features, thus resulting in no significant reduction in the number of features chosen. The paper does not clearly mention how many features are retained by the LASSO algorithm.

### (b) Some recent algorithms and open problems

Next, we discuss several versions of the problem formulation in (2.3) corresponding to diverse choices of the penalty norm R, culminating in some open problems that are relevant to biological applications. The ‘pure’ LASSO algorithm tries to choose as few distinct features as possible in the regressor. However, it may be worthwhile to partition the set of features N={1,…,n} into *g* groups *G*_1_,…,*G*_*g*_, and then choose a regressor that selects elements from as few distinct groups as possible, without worrying about the number of features chosen. This is achieved by the so-called group LASSO (GL) algorithm introduced in Bakin [23] and Lin & Zhang [24]. Let *n*_*l*_:=|*G*_*l*_| for *l*=1,…,*g*. In the grouped LASSO algorithm, the objective function is

2.7 $$J_{\mathrm{GL}}=\sum_{i=1}^{m}{\left(x^{i}w-\theta -y_{i}\right)}^{2}+\lambda \sum_{l=1}^{g}\sqrt{n_{l}}∥w_{G_{l}}{∥}_{2},$$

where $w_{G_{l}}\in R^{n}$ is determined from *w* by setting *w*_*j*_=0 for all *j*∉*G*_*l*_. It is clear that, depending on the relative sizes of the various groups, one weight vector can have more non-zero components than another, and yet the number of distinct groups to which these non-zero components belong can be smaller. In the limiting case, if the number of groups is taken as *n* and each group is taken to consist of a singleton set, then the grouped LASSO reduces to the standard LASSO algorithm. A further variation is the so-called sparse GL (SGL) algorithm introduced in Friedman *et al.* [25] and Simon *et al.* [26], where the objective is simultaneously to choose features from as few distinct groups as possible, and within the chosen groups, choose as few features as possible. The objective function in the SGL algorithm is

2.8 $$J_{\mathrm{SGL}}=\sum_{i=1}^{m}{\left(x^{i}w-\theta -y_{i}\right)}^{2}+\lambda \sum_{l=1}^{g}[(1-\mu )∥w_{G_{l}}{∥}_{1}+\mu ∥w_{G_{l}}{∥}_{2}],$$

where as always *μ*∈[0,1].

**Figure 1.**
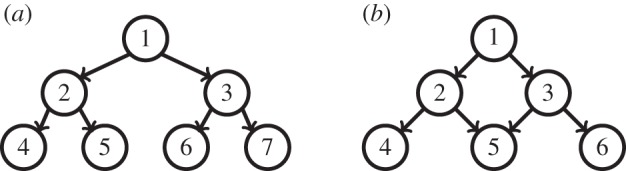
Two regulatory networks. (*a*) A network without overlapping groups. (*b*) A network with overlapping groups.

The above formulations of the GL and SGL norms are based on the assumption that the various groups do not overlap. However, in some biological applications it makes sense to permit overlapping group decompositions. Specifically, at a first level of approximation a GRN can be modelled as a directed acyclic graph, wherein the root nodes can be interpreted as master regulator genes, and directed paths can be interpreted as biological pathways. In such a case, one seeks to explain the available data, not by choosing the fewest number of *genes* but rather by the fewest number of *pathways*. To illustrate, consider the baby example shown in figure 1, where gene 1 is a master regulator, while genes 2–7 are regulated genes. Some are regulated directly by a master regulator gene, whereas others are indirectly regulated. In figure 1*a*, there are four pathways, namely

$$G_{1}=\{1,2,4\},\;G_{2}=\{1,2,5\},\;G_{3}=\{1,3,6\}\;\mathrm{and}\;G_{4}=\{1,3,7\},$$

whereas in figure 1*b* there are also four pathways, namely

$$G_{1}=\{1,2,4\},\;G_{2}=\{1,2,5\},\;G_{3}=\{1,3,5\}\;\mathrm{and}\;G_{4}=\{1,3,6\}.$$

Ideally, we would like to choose a set of features that intersect with as few pathways as possible. We will return to this example after presenting available theories for sparse regression with overlapping groups.

To date, various versions of group or SGL with overlapping groups have been proposed. As before, let *G*_1_,…,*G*_*g*_ be subsets of N={1,…,n}, but now *without* the assumption that the groups are pairwise disjoint. The penalty-augmented optimization problems are the same as in (2.7) and (2.8), respectively; however, the objective functions are now referred to as *J*_GLO_ and *J*_SGLO_ to suggest (sparse) GL with overlap. For the case of overlapping groups, the theory developed in Negabhan *et al.* [16] continues to apply so long as the penalty terms in (2.7) and (2.8), respectively, are ‘decomposable’. The most general results available to date address the case where the groups are ‘tree structured’, that is,

2.9 $$G_{i}\cap G_{j}\neq \emptyset \Rightarrow \{G_{i}\subseteq G_{j}\text{ or }G_{j}\subseteq G_{i}\}.$$

See, for example, Obozinski *et al.* [27] and Jenetton *et al.* [28].

Now, if we examine the groups associated with the network in figure 1*a*, it is obvious that (2.9) is not satisfied. However, there is a slight modification that would permit (2.9) to hold, namely to drop the root node and retain only the successors. Thus, the various groups are

$$\begin{array}{rl} G_{1} & =\{4\},\;G_{2}=\{5\},\;G_{3}=\{6\},\;G_{4}=\{7\},\;G_{5}=\{2,4\},\\ G_{6} & =\{2,5\},\;G_{7}=\{3,6\}\;\mathrm{and}\;G_{8}=\{3,7\}. \end{array}$$

However, there is no way of modifying the groups so as to ensure that (2.9) holds for the network in figure 1*b*. The reason is easy to see. The ‘tree structure’ assumption (2.9) implies that there is only one path between every pair of nodes. But this is clearly not true in figure 1*b*, because there are two distinct paths from node 1 to node 5. Moreover, a little thought would reveal that that the assumption of tree-structured groups does not really permit truly overlapping groups. In particular, if (2.9) holds, then the collection of sets {*G*_1_,…,*G*_*g*_} can be expressed as a union of chains in the form

$$G_{11}\subseteq \cdots \subseteq G_{1g_{1}},\ldots ,G_{s1}\subseteq \cdots \subseteq G_{sg_{s}},$$

where the ‘maximal’ sets *G*_*ig*_*i*__ are pairwise disjoint once duplicates are removed, and together span the total feature set N={1,…,n}. Now, in a biological network, it makes no sense to impose a condition that there must be only one path between every pair of nodes. Therefore, the problem of defining a decomposable norm penalty for inducing other types of sparsity besides tree structure, especially the types of sparsity that are consistent with biology, is still open.

We conclude this section with a new algorithm and its application to sparse regression. This represents joint work with Mehmet Eren Ahsen and will be presented in more complete form elsewhere. A special case of SGL is obtained by choosing just one group, which perforce has to equal N, so that

2.10 $$J_{\mathrm{MEN}}=\sum_{i=1}^{m}{\left(x^{i}w-\theta -y_{i}\right)}^{2}+\lambda [(1-\mu )∥w{∥}_{1}+\mu ∥w{∥}_{2}].$$

Of course, as the entire index set N is chosen as one group, there is nothing ‘sparse’ about it. Note that the only difference between (2.10) and (2.5) is that the ℓ_2_-norm is *not* squared in the former. For this reason, the above approach is called the ‘modified elastic net’ or MEN algorithm. Unlike in EN, the penalty (or constraint) term in MEN is a norm, being a convex combination of the ℓ_1_- and ℓ_2_-norms. In several examples, the MEN algorithm appears to combine the accuracy of EN with the sparsity of LASSO. It is relatively easy to prove an analogue of theorem 2.1 for the MEN algorithm. That is, unlike in LASSO but as in EN, MEN assigns nearly equal weights to highly correlated features. But further theoretical analysis remains to be carried out.

**Figure 2.**
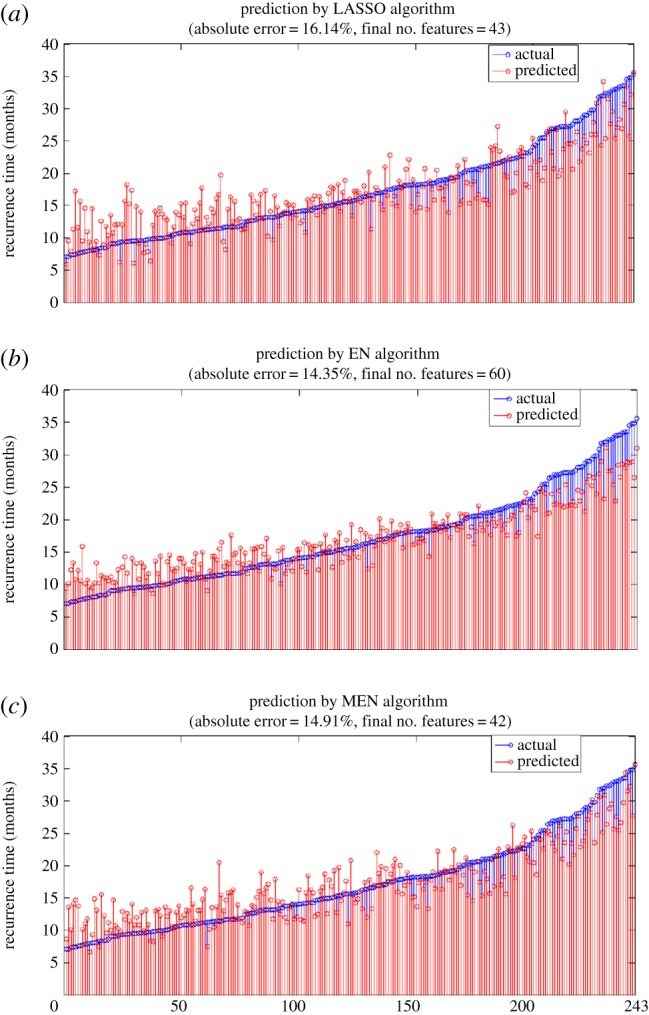
Predicted versus actual times to tumour recurrence in 243 ovarian cancer patients. The results for the LASSO algorithm are in (*a*), those for the EN algorithm are in (*b*) and those for the MEN algorithm are in (*c*). (Online version in colour.)

The MEN algorithm was applied to the TCGA ovarian cancer data [29] to predict the time to tumour recurrence. Specifically, both times to tumour recurrence as well as expression levels for 12 042 genes are available for 283 patients. Out of these, 40 patients whose tumours recurred before 210 days or after 1095 days were excluded from the study as being ‘extreme’ cases. The remaining 243 samples were analysed using MEN with recursive feature elimination (RFE). The results are shown in figure 2. The number of features and the average percentage error in absolute value are shown in table 1.

**Table 1.** Comparison of three algorithms on TCGA ovarian cancer data on time to tumour recurrence, with extreme cases excluded.

algorithm	no. features	average perc. error (%)
LASSO	43	16.14
EN	60	14.35
MEN	42	14.91

## 3. Compressed sensing

In recent years, there have been several results that are grouped under the general heading of ‘compressed sensing’ or ‘compressive sensing’. Both expressions are in use, but ‘compressed sensing’ is used in this paper. The problem can be roughly stated as follows: suppose $x\in R^{n}$ is an unknown vector but with known structure; is it possible to determine *x* either exactly or approximately by taking *m*≪*n* linear measurements of *x*? The area of research that goes under this broad heading grew spectacularly during the first decade of the new millennium.^5^ As summarized in the introduction of the paper [31], the impetus for recent work in this area was the desire to find algorithms for data compression that are ‘universal’ in the sense of being non-adaptive (i.e. do not depend on the data). In the original papers in this area, the results and proofs were a mixture of sampling, signal transformation (time domain to frequency domain and vice versa), randomness, etc. However, as time went on, the essential ingredients of the approach were identified, thus leading to a very streamlined theory that clearly transcends its original application domains of image and signal processing.

Image processing is one of the potential (and actually realized) applications of compressed sensing, and, as such, the theory has already been applied to the processing of biological images. Other than this, there do not appear to be any applications of the theory to cancer biology. Perhaps this can be attributed to the relative newness of the subject. The motivation for discussing compressed sensing theory in this paper is the following: whether it is in compressed sensing or in computational biology, one searches for a relatively simple explanation of the observations. Therefore, it may *potentially* be possible to borrow some of the basic ideas from compressed sensing theory and adapt them to problems in cancer biology. Compressed sensing theory *as it currently stands* cannot directly be applied to the analysis of biological datasets, because the fundamental assumption in compressed sensing theory is that *one is able to choose* the so-called measurement matrix, called *A* throughout this paper. Note that, in statistics, the matrix *A* is often referred to as the ‘design’ matrix. However, in biological (and other) applications, the measurement matrix is given, and one does not have the freedom to change it. Nevertheless, the developments in this area are too important to be ignored by computational biologists. The hope is that, by understanding the core arguments of compressed sensing theory, the computational biology community would be able to adapt the theory for its application domain. In parallel, those who are well versed in compressed sensing theory can start thinking about how the basic arguments can be modified to the case where the measurement matrix is specified, and cannot be chosen.

The major developments in this area are generally associated with the names of Candès, Donoho, Romberg and Tao, though several other researchers have also made important contributions. See Donoho [31] for one of the earliest comprehensive papers, as well Donoho [32], Candés [33], Candés & Tao [34,35], Candés & Plan [36], Romberg [37] and Cohen *et al.* [38]. The survey paper [30] and a recent paper [16] contain a wealth of bibliographic references that can be followed up by interested readers.

We begin by introducing some notation. Suppose *m*,*n*,*k* are given integers, with *n*≥2*k*. For convenience, we denote the set {1,…,*n*} by N throughout. For a given vector $x\in R^{n}$, let supp(*x*) denote its support, that is, supp(*x*)={*i*:*x*_*i*_≠0}. Let, $\Sigma_{k}=\{x\in R^{n}:|\text{supp}(x)|\leq k\}$. Thus, *Σ*_*k*_ denotes the set of ‘*k*-sparse’ vectors in $R^{n}$, or, in other words, the set of *n*-dimensional vectors that have *k* or fewer non-zero components. For each vector $x\in R^{n}$, integer *k*<*n* and norm ∥⋅∥ on $R^{n}$, the symbol *σ*_*k*_(*x*,∥⋅∥) denotes the distance from *x* to *Σ*_*k*_, that is,

$$\sigma_{k}(x,∥\cdot ∥)=\inf\{∥x-z∥:z\in \Sigma_{k}\}.$$

The quantity *σ*_*k*_(*x*,∥⋅∥) is called the ‘sparsity measure’ of the vector *x* of order *k* with respect to the norm ∥⋅∥. It is obvious that *σ*_*k*_(*x*,∥⋅∥) depends on the underlying norm. However, if ∥⋅∥ is one of the ℓ_*p*_-norms, then it is easy to compute *σ*_*k*_(*x*,∥⋅∥). Specifically, given *k*, let *Λ*_0_ denote the index set corresponding to the *k*-largest components of *x* in magnitude, and let $x_{\Lambda_{0}^{c}}$ denote the vector that results by replacing the components of *x* in the set *Λ*_0_ by zeros. (It is convenient to think of *x*_*Λ*^*c*^_ as an element of $R^{n}$ rather than an element of $R^{n-k}$.) Then, whenever p∈[1,∞], it is easy to see that

$$\sigma_{k}(x,∥\cdot {∥}_{p})=∥x_{\Lambda_{0}^{c}}{∥}_{p}.$$

Next, the so-called ‘restricted isometry property’ (RIP) is introduced. Note that, in some cases, the RIP can be replaced by a weaker property known as the ‘null space property’ [38]. However, the objective of this paper is *not* to present the most general results, but rather to present reasonably general results that are easy to explain. So the exposition below is confined to the RIP.

**Definition 3.1**

Suppose $A\in R^{m\times n}$. We say that *A* satisfies the *RIP* of order *k* with constant *δ*_*k*_ if

3.1 $$(1-\delta_{k})∥u{∥}_{2}^{2}\leq \langle u,Au\rangle \leq (1+\delta_{k})∥u{∥}_{2}^{2},\;\forall u\in \Sigma_{k}.$$

So the matrix *A* has the RIP of order *k* with constant 1−*δ*_*k*_ if the following property holds: for every choice of *k* or fewer columns of *A* (say the columns in the set J⊆N, where |*J*|≤*k*), the spectrum of the symmetric matrix $A_{J}^{t}A_{J}$ lies in the interval [1−*δ*_*k*_,1+*δ*_*k*_], where $A_{J}\in R^{m\times |J|}$ denotes the submatrix of *A* consisting of all rows and the columns corresponding to the indices in *J*.

If integers *n*,*k* are specified, the integer *m* has to be sufficiently large in order for the matrix *A* to satisfy the RIP.

**Theorem 3.2 (Davenport et al. [30, theorem 1.4])**

*Suppose* $A\in R^{m\times n}$ *satisfies the RIP or order 2k with constant δ*_2*k*_*∈(0,1/2]. Then,*

3.2 $$m\geq ck\log\left(\frac{n}{k}\right)=ck(\log n-\log k),$$

*where*

$$c=\frac{1}{2\log(\sqrt{24}+1)}\approx 0.28.$$

Next, we state some of the main known results in compressed sensing. The theorem statement below corresponds to Candès [33, theorem 1.2] and Davenport *et al.* [30, theorem 1.9].

**Theorem 3.3**

*Suppose* $A\in R^{m\times n}$ *satisfies the RIP of order δ*_2*k*_ *with constant* $\delta_{2k}<\sqrt{2}-1,$ *and that y=Ax+η for some* $x\in R^{n}$ *and* $\eta \in R^{m}$ *with ∥η∥*_2_*≤ϵ. Define*

3.3 $$\hat{x}=\underset{z\in R^{n}}{\mathrm{argmin}}∥z{∥}_{1}\;\text{ s.t. }∥y-Az{∥}_{2}\leq \epsilon .$$

*Then,*

3.4 $$∥\hat{x}-x{∥}_{2}\leq C_{0}\frac{\sigma_{k}(x,∥\cdot {∥}_{1})}{\sqrt{k}}+C_{2}\epsilon ,$$

*where*

3.5 $$C_{0}=2\frac{1+(\sqrt{2}-1)\delta_{2k}}{1-(\sqrt{2}+1)\delta_{2k}}\;\mathrm{and}\;C_{2}=\frac{4\sqrt{1+\delta_{2k}}}{1-(\sqrt{2}+1)\delta_{2k}}.$$

The formula for *C*_2_ is written slightly differently from that in Davenport *et al.* [30, theorem 1.9] but is equivalent to it.

**Corollary 3.4**

*Suppose* $A\in R^{m\times n}$ *satisfies the RIP of order* *δ*_2*k*_ *with constant* $\delta_{2k}<\sqrt{2}-1,$ *and that* *y*=*Ax*+*η* *for some* *x*∈*Σ*_*k*_ *and* $\eta \in R^{m}$ *with* ∥*η*∥_2_≤*ϵ*. *Define*

3.6 $$\hat{x}=\underset{z\in R^{n}}{\mathrm{argmin}}∥z{∥}_{1}\;\text{ s.t. }∥y-Az{∥}_{2}\leq \epsilon .$$

*Then*,

3.7 $$∥\hat{x}-x{∥}_{2}\leq C_{2}\epsilon ,$$

*where* *C*_2_ *is defined in* (3.5).

**Corollary 3.5**

*Suppose* $A\in R^{m\times n}$ *satisfies the RIP of order* *δ*_2*k*_ *with constant* $\delta_{2k}<\sqrt{2}-1,$ *and that* *y*=*Ax* *for some* *x*∈*Σ*_*k*_. *Let*, $A^{-1}(y):=\{z\in R^{n}:y=Az\}$ *and define*

3.8 $$\hat{x}=\underset{z\in A^{-1}(y)}{\mathrm{argmin}}∥z{∥}_{1}.$$

*Then*, $\hat{x}=x$.

Both corollaries follow readily from the bound (3.4). Note that if *x*∈*Σ*_*k*_, then *σ*_*k*_(*x*,∥⋅∥_1_)=0. Thus, (3.4) implies that $∥\hat{x}-x{∥}_{2}\leq C_{2}\epsilon$ if there is measurement error, and $∥\hat{x}-x{∥}_{2}=0$, i.e. that $\hat{x}=x$, if there no measurement error.

Corollary 3.4 is referred to as the ‘near ideal’ property of the LASSO algorithm. Suppose that *x*∈*Σ*_*k*_ so that *x* is *k*-sparse. Let *S* denote the support of *x*, and let $A_{S}\in R^{m\times |S|}$ denote the submatrix of *A* consisting of the columns corresponding to indices in *S*. If an ‘oracle’ knew not only the size of *S*, but the set *S* itself, then the oracle could compute $\hat{x}$ as

$${\hat{x}}_{\mathrm{oracle}}={\left(A_{S}^{T}A_{S}\right)}^{-1}A_{S}^{T}y=x+{\left(A_{S}^{T}A_{S}\right)}^{-1}A_{S}^{T}\eta .$$

Then, the error would be

$$∥{\hat{x}}_{\mathrm{oracle}}-x{∥}_{2}=∥{\left(A_{S}^{T}A_{S}\right)}^{-1}A_{S}^{T}\eta {∥}_{2}\leq \text{const}\cdot \epsilon$$

for some appropriate constant. On the other hand, if *x*∈*Σ*_*k*_, then *σ*_*k*_(*x*,∥⋅∥_1_)=0, and the right-hand side of (3.4) reduces to (3.7), that is,

$$∥\hat{x}-x{∥}_{2}\leq C_{2}\epsilon .$$

The point therefore is that, if the matrix *A* satisfies RIP, and the constant *δ*_2*k*_ satisfies the ‘compressibility condition’ $\delta_{2k}<\sqrt{2}-1$, then the mean-squared error of the solution to the optimization problem (3.6) is bounded by a fixed (or ‘universal’) constant times the error bound achieved by an ‘oracle’ that knows the support of *x*.

It should be noted that there is a parallel, and closely related, set of papers that study the following problem: given a matrix $A\in R^{m\times n}$, a feature vector *x* that is known to be *k*-sparse but otherwise unknown, and a random measurement error *w* assuming values in $R^{m}$, suppose one is given the noise-corrupted measurement *y*=*Ax*+*w*. To recover *x* from *y*, one solves the minimization problem

3.9 $$x^{*}=\underset{z\in R^{n}}{\mathrm{argmin}}∥y-Az{∥}_{2}^{2}+\lambda ∥z{∥}_{1},$$

where *l* is a user-specified penalty weight. What, if any, is the relationship between x* and *x*? It is easy to see that the objective function in (3.9) is just the Lagrangian associated with the constrained objective function in (3.3). Specifically, if λ is sufficiently large, then large values of ∥*z*∥_1_ are penalized, and the problem in (3.9) begins to resemble that in (3.3). Of course, the bound *ϵ* on the magnitude of the noise is not present in the problem formulation (3.9). In Candès & Plan [36] and Negabhan *et al.* [16], the above problem is analysed, and probabilistic (with respect to the random noise *w*) bounds analogous to (3.7) are derived. Indeed, [16] contains a very general theory wherein the ℓ_2_-norm of *y*−*Az* is replaced by an arbitrary convex function, and the ℓ_1_-norm is replaced by any *decomposable* norm.

The advantage of the above theorem statements, which are taken from Candès [33] and Davenport *et al.* [30], is that the role of various conditions is clearly delineated. For instance, the construction of a matrix $A\in R^{m\times n}$ that satisfies the RIP is usually achieved by some randomized algorithm. In Candès & Tao [34, theorem 1.5], such a matrix is constructed by taking the columns of *A* to be samples of i.i.d. Gaussian variables. In Achlioptas [39], Bernoulli processes are used to construct *A*, which has the advantage of ensuring that all elements *a*_*ij*_ have just three possible values, namely 0,+1,−1. A simple proof that the resulting matrices satisfy the RIP with high probability is given in Baraniuk *et al.* [40]. Neither of these construction methods is guaranteed to generate a matrix *A* that satisfies RIP. Rather, the resulting matrix *A* satisfies RIP with some probability, say ≥1−*γ*_1_. The probability *γ*_1_ that the randomized method may fail to generate a suitable *A* matrix can be bounded using techniques that have nothing to do with the above theorem. Similarly, in case the measurement matrix *A* satisfies the RIP but the measurement noise *η* is random, then it is obvious that theorem 3.3 holds with probability ≥1−*γ*_2_, where *γ*_2_ is a bound on the tail probability $\mathrm{Pr}\{∥\eta {∥}_{2}>\epsilon \}$. Again, the problem of bounding this tail probability has nothing to do with theorem 3.3. By combining both estimates, it follows that if the measurement matrix *A* is generated through randomization, and if the measurement noise is also random, then theorem 3.3 holds with probability ≥1−*γ*_1_−*γ*_2_.

Observe that the optimization problem (3.6) is

$$\min_{z}∥z{∥}_{1}\;\text{ s.t. }∥y-Az{∥}_{2}\leq \epsilon .$$

This raises the question as to whether the ℓ_1_-norm can be replaced by some other norm ∥⋅∥_P_ that induces some other form of sparsity, for example group sparsity. If some other norm is used in place of the ℓ_1_-norm, does the resulting algorithm display near-ideal behaviour, as does LASSO? In other words, is there an analogue of theorem 3.3 if ∥⋅∥_1_ is replaced by another penalty ∥⋅∥_P_? In joint work with Ahsen [41], the author has proved a very general theorem to the following effect: whenever the penalty norm is ‘decomposable’ and the measurement matrix *A* satisfies a ‘group RIP’, the corresponding algorithm has near-ideal behaviour provided a ‘compressibility condition’ is satisfied. The result is described in brief.

Let $G=\{G_{1},\ldots ,G_{g}\}$ be a partition of N={1,…,n}. This implies that the sets *G*_*i*_ are pairwise disjoint. If *S*⊆{1,…,*g*}, define *G*_S_:=∪_*i*∈*S*_*G*_*i*_. Let *k* be some integer such that $k\geq \max_{i}|G_{i}|$. A subset Λ⊆N is said to be **S*-group *k*-sparse* if *Λ*=*G*_S_ and |*G*_S_|≤*k*, and *group *k*-sparse* if it is *S*-group *k*-sparse for some set *S*⊆{1,…,*g*}. The symbol $\mathrm{GkS}\subseteq 2^{N}$ denotes the collection of group *k*-sparse sets.

Suppose $∥\cdot {∥}_{P}:R^{n}\to R_{+}$ is some norm. The next definition builds on an earlier definition from Negabhan *et al.* [16].

**Definition 3.6**

The norm ∥⋅∥_P_ is *decomposable* with respect to the partition G if the following is true: whenever $u,v\in R^{n}$ are group *k*-sparse with support sets *Λ*_*u*_⊆*G*_*S*_1__, *Λ*_*v*_⊆*G*_*S*_2__ and the sets *S*_1_,*S*_2_ are disjoint, it is true that

3.10 $$∥u+v{∥}_{P}=∥u{∥}_{P}+∥v{∥}_{P}.$$

By adapting the arguments in Negabhan *et al.* [16], it can be shown that the GL norm used in (2.7), namely

$$∥x{∥}_{\mathrm{GL}}:=\sum_{l=1}^{g}\sqrt{n_{l}}∥x_{G_{l}}{∥}_{2},$$

and the SGL norm used in (2.8), namely

$$∥x{∥}_{\mathrm{SL}}:=\sum_{l=1}^{g}[(1-\mu )∥x_{G_{l}}{∥}_{1}+\mu ∥x_{G_{l}}{∥}_{2}],$$

are both decomposable.

Next, the notion of RIP is extended to groups.

**Definition 3.7**

A matrix $A\in R^{m\times n}$ is said to *satisfy the group RIP of order *k* with constants ${\underline{\rho }}_{k},{\bar{\rho }}_{k}$* if

3.11 $$0<{\underline{\rho }}_{k}\leq \min_{\Lambda \in \mathrm{GkS}}\;\min_{\text{supp}(z)\subseteq \Lambda }\frac{∥Az{∥}_{2}^{2}}{∥z{∥}_{2}^{2}}\leq \max_{\Lambda \in \mathrm{GkS}}\;\max_{\text{supp}(z)\subseteq \Lambda }\frac{∥Az{∥}_{2}^{2}}{∥z{∥}_{2}^{2}}\leq {\bar{\rho }}_{k}.$$

We define $\delta_{k}:=({\bar{\rho }}_{k}-{\underline{\rho }}_{k})/2$ and introduce some constants

3.12 $$c:=\min_{\Lambda \in \mathrm{GkS}}\;\min_{x_{\Lambda }\neq 0}\frac{∥x_{\Lambda }{∥}_{P}}{∥x_{\Lambda }{∥}_{2}}\;\mathrm{and}\;d:=\max_{\Lambda \in \mathrm{GkS}}\;\max_{x_{\Lambda }\neq 0}\frac{∥x_{\Lambda }{∥}_{P}}{∥x_{\Lambda }{∥}_{2}}.$$

With these definitions, the following theorem can be proved.

**Theorem 3.8**

*Suppose* $A\in R^{m\times n}$ *satisfies the group RIP property of order 2k with constants* $({\underline{\rho }}_{2k},{\bar{\rho }}_{2k}),$ *respectively, and let* $\delta_{2k}=({\bar{\rho }}_{2k}-{\underline{\rho }}_{2k})/2$*. Suppose* $x\in R^{n}$ *and that y=Ax+η, where ∥η∥*_2_*≤ϵ. Suppose that the norm ∥⋅∥*_P_ *is decomposable, and define*

3.13 $$\hat{x}=\underset{z\in R^{n}}{\mathrm{argmin}}∥z{∥}_{P}\;\text{ s.t. }∥y-Az{∥}_{2}\leq \epsilon .$$

*Suppose that the compressibility condition*

3.14 $$\delta_{2k}<\frac{c{\underline{\rho }}_{k}}{d}$$

*is satisfied. Then,*

3.15 $$∥\hat{x}-x{∥}_{P}\leq \frac{2}{1-r}[2(1+r)\sigma +\zeta \epsilon ]$$

*and*

3.16 $$∥\hat{x}-x{∥}_{2}\leq \frac{2}{c(1-r)}[2(1+r)\sigma +\zeta \epsilon ],$$

*where σ is shorthand for the sparsity index*

3.17 $$\sigma =\sigma_{k,G}(x,∥\cdot {∥}_{P}):=\min_{\Lambda \in \mathrm{GkS}}∥x-x_{\Lambda }{∥}_{P}=\min_{\Lambda \in \mathrm{GkS}}∥x_{\Lambda_{0}^{c}}{∥}_{P}$$

*and*

3.18 $$r:=\frac{\delta_{2k}d}{c{\underline{\rho }}_{k}}\;\mathrm{and}\;\zeta :=\frac{2d\sqrt{{\bar{\rho }}_{k}}}{{\underline{\rho }}_{k}},$$

*and c,d are defined in (*3.12*).*

In the above theorem, (3.14) replaces the compressibility condition $\delta_{2k}<\sqrt{2}-1$ of theorem 3.3. The resemblance of (3.16) to (3.4) is obvious. Consequently, (3.16) can be readily interpreted as stating that minimizing the decomposable norm ∥⋅|∥_P_ leads to near-ideal behaviour.

## 4. Classification methods

The basic problem of classification can be stated as follows: suppose we are given a collection of labelled vectors (*x*^*i*^,*y*_*i*_),*i*=1,…,*m*, where each $x^{i}\in R^{n}$ is viewed as a row vector and each *y*_*i*_∈{−1,1}. For future use, define

$$M_{1}:=\{i:y_{i}=1\}\;\mathrm{and}\;M_{2}=\{i:y_{i}=-1\}.$$

The objective of (two-class) classification is to find a *discriminant function* $f:R^{n}\to R$ such that *f*(*x*^*i*^) has the same sign as *y*_*i*_ for all *i*, or equivalently *y*_*i*_⋅sign ( *f*(*x*^*i*^))=1 for all *i*. In the present context, the objective is not merely to find such a discriminant function, but, rather, to find one that uses relatively few features.

In many ways, classification is an easier problem than regression, because the sole criterion is that the discriminant function *f*(*x*^*i*^) should have the same sign as the label *y*_*i*_ for each *i*. Thus, if *f* is a discriminant function, so is *αf* for every positive constant *α*, and, more generally, so is any function *ϕ*( *f*) whenever *ϕ* is a so-called ‘first- and third-quadrant function’, i.e. where *ϕ*(*u*)>0 when *u*>0 and *ϕ*(*u*)<0 when *u*<0. This gives us great latitude in choosing a discriminant function.

### (a) The ℓ_2_-norm support vector machine

This section is devoted to the well-known SVM, first introduced in Cortes & Vapnik [42], which is among the most successful and most widely used tools in machine learning. To distinguish this algorithm from its variants, it is referred to here as the ℓ_2_-norm SVM, for reasons that will become apparent.

A given set of labelled vectors $\left\{(x^{i},y_{i}),x^{i}\in R^{n},y_{i}\in \{-1,1\}\right\}$ is said to be **linearly separable** if there exist a ‘weight vector’ $w\in R^{n}$ (viewed as a column vector) and a ‘threshold’ θ∈R such that *f*(*x*)=*xw*−*θ* serves as a discriminant function. Equivalently, the dataset is linearly separable if there exist a weight vector $w\in R^{n}$ and a threshold θ∈R such that

$$x^{i}w>\theta \;\forall i\in M_{1}\;\mathrm{and}\;x^{i}w<\theta \;\forall i\in M_{2}.$$

To put it yet another way, given a weight *w* and a threshold *θ*, define H=H(w,θ) by

$$H:=\{x\in R^{n}:xw-\theta =0\}.H_{+}:=\{x\in R^{n}:xw-\theta >0\},\;H_{-}:=\{x\in R^{n}:xw-\theta <0\}.$$

The dataset is linearly separable if there exists a hyperplane H such that $x^{i}\in H_{+}\;\forall i\in M_{1}$ and $x^{i}\in H_{-}\;\forall i\in M_{2}$.

**Figure 3.**
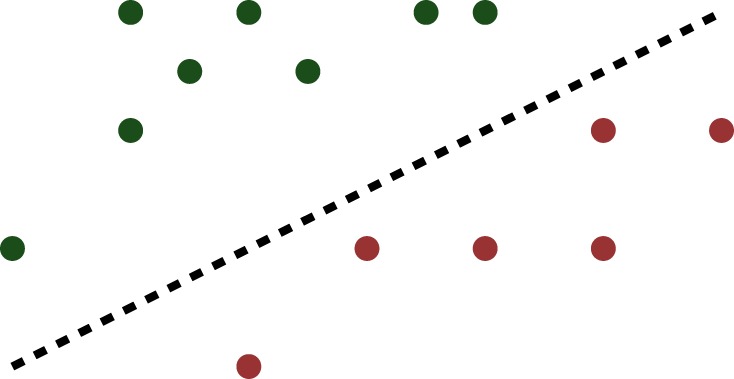
A linearly separable dataset. (Online version in colour.)

The situation can be depicted as in figure 3*a*, in which the dots on either side of the dashed line represent the two classes. It is clear that linear separability is not affected by swapping the class labels.

**Figure 4.**
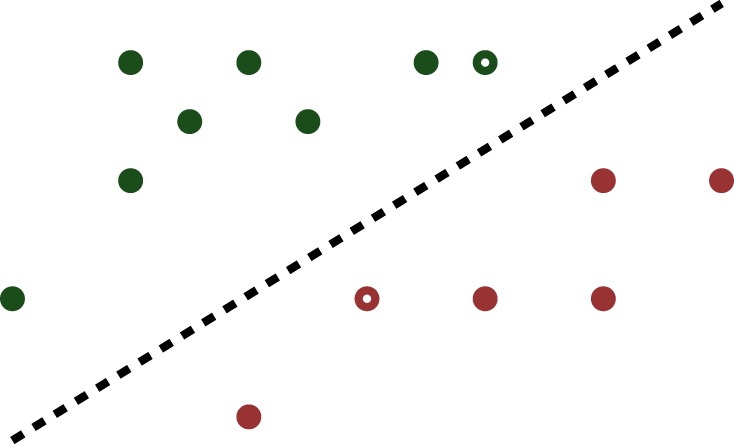
Optimal separating hyperplane. (Online version in colour.)

It is easy to see that, if there exists *one* hyperplane that separates the two classes, there exist *infinitely many* such hyperplanes. The question therefore arises as to which of these choices is the best. The SVM introduced in Cortes & Vapnik [42] chooses the separating hyperplane such that *the nearest point to the hyperplane within each class is as far as possible from it*. In the original SVM formulation, the distance to the hyperplane is measured using the Euclidean or ℓ_2_-norm. To illustrate the concept, the same dataset as in figure 3 is shown again in figure 4, with the ‘optimal’ separating hyperplane, and the closest points to it within the two classes shown as hollow circles.

In symbols, the SVM is obtained by solving the following optimization problem:

$$\max_{w,\theta }\min_{i}\underset{v\in H}{\inf}∥v-x^{i}∥.$$

An equivalent formulation of the SVM is obtained by observing that the distance of the separating hyperplane to the nearest points is given by *c*/∥*w*∥, where

$$c:=\min_{i\in M_{1}}|y_{i}(x^{i}w-\theta )|=\min_{i\in M_{2}}|y_{i}(x^{i}w-\theta )|,$$

where the equality of the two terms follows from the manner in which the separating hyperplane is chosen. Moreover, the optimal hyperplane is invariant under scale change, that is, multiplying *w* and *θ* by a positive constant. Therefore, there is no loss of generality in taking the constant *c* to equal one. With this rescaling, the problem at hand becomes the following:

4.1 $$\min_{w}∥w∥\;\text{ s.t. }x^{i}w\geq 1\;\forall i\in M_{1}\;\mathrm{and}\;x^{i}w\leq -1\;\forall i\in M_{2}.$$

This is the manner in which the SVM is implemented nowadays in most software packages.

The original SVM formulation presupposes that the dataset is linearly separable. It is easy to determine whether or not a given dataset is linearly separable, because that is equivalent to the feasibility of a linear programming problem. This naturally raises the question of what is to be done in case the dataset is not linearly separable. One way to approach the problem is to choose a hyperplane that misclassifies the fewest number of points. While appealing, this approach is impractical, because it is known that this problem is NP-hard; see Höffgen *et al.* [43] and Natarajan [15]. A tractable approach is to replace this problem by its convex relaxation. We will return to this issue when we discuss ℓ_1_-norm SVMs.

An alternative approach to guarantee that the data are linearly separable can be obtained using Vapnik–Chervonenkis theory [44,45]. Suppose that the *n* vectors *x*_1_,…,*x*_*n*_ do not lie on an (*p*−1)-dimensional hyperplane in $R^{p}$. In such a case, whenever *p*≥*n*−1, the dataset is linearly separable for *every one* of the 2^*n*^ ways of assigning labels to the *n* vectors. This result suggests that, if a given dataset is not linearly separable, it can be made so by increasing the dimension of the data vectors *x*^*i*^, for instance, by including not just the original components but also their higher powers. This is the rationale behind so-called ‘higher order’ SVMs, or, more generally, kernel-based classifiers (e.g. Cristianini & Shawe-Taylor [46] and Schölkopf & Smola [47]).

**Figure 5.**
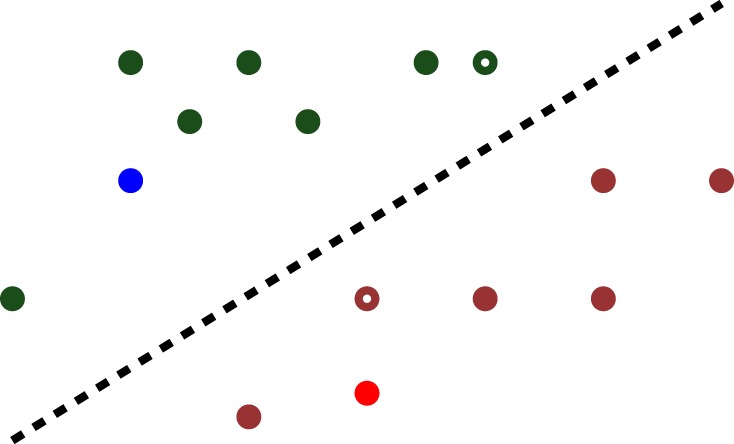
Insensitivity of optimal separating hyperplane to additional samples. (Online version in colour.)

If the norm in (4.1) is the ℓ_2_-norm, then the minimization problem (4.1) is a quadratic programming problem, which can be solved efficiently for extremely large datasets. Moreover, the introduction of new data points does not alter the optimal hyperplane, unless one of the new data points is closer to the hyperplane than the earlier closest points. This is illustrated in figure 5, which contains exactly the same vectors as in figure 4, plus two more. The optimal hyperplane remains the same. For all these reasons, the SVM offers a very attractive approach to finding a classifier in situations where the number of features is smaller than the number of samples. On the other hand, generically the optimal weight vector has all non-zero components, which is undesirable when the number of samples *m* is too large, even if *m*<*n*. To overcome this problem, an approach known as RFE is suggested in Guyon *et al.* [48]. This consists of solving the SVM problem (4.1), identifying the component of the weight vector with the smallest magnitude, discarding it, re-solving the problem and repeating. Though it is claimed in Guyon *et al.* [48] that the method works well on a leukaemia dataset, in general RFE applied to the traditional ℓ_2_-norm SVM displays rather erratic behaviour.

### (b) The ℓ_1_-norm support vector machine

As we have seen, in biological applications, the number of features (the dimension of the vectors *x*^*i*^) is a few orders of magnitude larger than the number of samples (the number of vectors). In such a case, because of the results in Wenocur & Dudley [44], linear separability is not an issue. However, in general, *every component* of the optimal weight vector *w* is non-zero. This means that a classifier uses every single feature in order to discriminate between the classes. Clearly, this is undesirable. The original SVM formulation presupposes that the dataset is linearly separable. If the data are not linearly separable, then as shown in Höffgen *et al.* [43] and Natarajan [15], the problem of finding a hyperplane that misclassifies the fewest points is NP-hard. An alternative approach is to formulate a convex relaxation of this NP-hard problem by introducing slack variables into the constraints in (4.1), and then minimizing an appropriate norm of the vector of slack variables. Finally, in many problems, the consequences of misclassification might not be symmetric. A false positive (labelling a sample as positive when in fact it is negative) might have far more, or far less, severe consequences than a false negative. In this section, we present a problem formulation that addresses all of these issues. This problem formulation combines the ideas in two papers, namely [49,50].

If we choose a particular norm ∥⋅∥ to measure distances in ‘feature space’, then distances in ‘weight space’ should be measured using the so-called **dual norm**, defined by

$$∥w{∥}_{d}:=\underset{∥x∥\leq 1}{\sup}|xw|.$$

In particular, if we measure distances in feature space using the ℓ_1_-norm, then distances in weight space should be measured using its dual, which is the $\ell_{\infty }$-norm. With this observation, the problem can be formulated as follows:

4.2 $$\begin{array}{rl} & \min_{w,\theta ,y,z}(1-\lambda )\left[\sum_{i=1}^{m_{1}}y_{i}+\sum_{i=1}^{m_{2}}z_{i}\right]+\lambda \max_{1\leq i\leq n}|w_{i}|\;\text{ s.t. }\\ & x^{i}w-\theta +y_{i}\geq 1\;\forall i\in M_{1},\;x^{i}w-\theta -z_{i}\leq -1\;\forall i\in M_{2},\\ & y\geq 0_{m_{1}}\;\mathrm{and}\;z\geq 0_{m_{2}}. \end{array}\}$$

This can be converted to

4.3 $$\begin{array}{rl} & \min_{w,\theta ,y,z}(1-\lambda )\left[\sum_{i=1}^{m_{1}}y_{i}+\sum_{i=1}^{m_{2}}z_{i}\right]+\lambda v\;\text{ s.t. }\\ & x^{i}w-\theta +y_{i}\geq 1\;\forall i\in M_{1},\;x^{i}w-\theta -z_{i}\leq -1\;\forall i\in M_{2},\\ & y\geq 0_{m_{1}},\;z\geq 0_{m_{2}}\;\mathrm{and}\;v\geq w_{i}\;\forall i,v\geq -w_{i}\;\forall i. \end{array}\}$$

This is clearly a linear programming problem. In this formulation, λ is a ‘small’ constant in (0,1), much closer to 0 than it is to 1. Suppose that the original dataset is linearly separable, and let w*,θ* denote a solution to the optimization problem in (4.1), where ∥*w*∥_*d*_ replaces ∥*w*∥. Then the choice

$$w=w^{*},\;\theta =\theta^{*},\;y=0_{m_{1}}\;\mathrm{and}\;z=0_{m_{2}}$$

is certainly *feasible* for the optimization problem (4.2). Moreover, if λ is sufficiently small, any reduction in ∥*w*∥_*d*_ achieved by violating the linear separation constraints (i.e. permitting some *y*_*i*_ or *z*_*i*_ to be positive rather than zero) is offset by the increase in the term (1−λ)∥(*y*,*z*)∥. It is therefore clear that, if the dataset is linearly separable, there exists a critical value λ_0_>0 such that, for all λ<λ_0_, the optimization problem (4.2) has (w*,*θ*,**0**_*m*_1__,**0**_*m*_2__) as a solution. On the other hand, the optimization problem (4.2) remains meaningful even when the data are not linearly separable.

The final aspect of the problem, as suggested in Veropoulos *et al.* [50], is to introduce a trade-off between false positives and false negatives. In this connection, it is worthwhile to recall the definitions of the accuracy, etc. of a classifier. Given a discriminant function *f*(⋅), define

$$C_{1}:=\{i\in M:f(x^{i})>0\}\;\mathrm{and}\;C_{2}:=\{i\in M:f(x^{i})<0\}.$$

Thus, $C_{1}$ consists of the samples that are assigned to class 1 by the classifier, while $C_{2}$ consists of the samples that are assigned to class 2. Then, this leads to the array shown below

$$\begin{array}{ccc} & C_{1} & C_{2}\\ M_{1} & \mathrm{TP} & \mathrm{FN}\\ M_{2} & \mathrm{FP} & \mathrm{TN} \end{array}$$

In the above array, the entries TP, FN, FP and TN stand for ‘true positive’, ‘false negative’, ‘false positive’ and ‘true negative’, respectively.

**Definition 4.1**

With the above definitions, we have

4.4 $$\begin{array}{rl} \mathrm{Se} & =\frac{\mathrm{TP}}{\mathrm{TP}+\mathrm{FN}}=\frac{|C_{1}\cap M_{1}|}{|M_{1}|}, \end{array}$$

4.5 $$\begin{array}{rl} \mathrm{Sp} & =\frac{\mathrm{TN}}{\mathrm{FP}+\mathrm{TN}}=\frac{|C_{2}\cap M_{2}|}{|M_{2}|} \end{array}$$

4.6 $$\begin{array}{rl} \mathrm{and}\;\;\;\mathrm{Ac} & =\frac{\mathrm{TP}+\mathrm{TN}}{\mathrm{TP}+\mathrm{TN}+\mathrm{FP}+\mathrm{FN}}=\frac{|C_{1}\cap M_{1}|+|C_{2}\cap M_{2}|}{|M_{1}|+|M_{2}|}, \end{array}$$

where Se, Sp and Ac stand for the *sensitivity*, *specificity* and *accuracy*, respectively.

All three quantities lie in the interval [0,1]. Moreover, accuracy is a convex combination of sensitivity and specificity. In particular,

$$\mathrm{Ac}=\mathrm{Se}\cdot \frac{|M_{1}|}{|M_{1}|+|M_{2}|}+\mathrm{Sp}\cdot \frac{|M_{2}|}{|M_{1}|+|M_{2}|}.$$

Therefore,

min{Se, Sp}≤Ac≤max{Se, Sp}.

Also, the accuracy of a classifier will be roughly equal to the sensitivity if $M_{1}$ is far larger than $M_{2}$, and roughly equal to the specificity if $M_{2}$ is far larger than $M_{1}$.

In many classification problems, the consequences of misclassification are not symmetric. To capture these kinds of considerations, another parameter *α*∈(0,1) is introduced, and the objective function in the optimization problem (4.2) is modified by making the substitution

$$\sum_{i=1}^{m_{1}}y_{i}+\sum_{i=1}^{m_{2}}z_{i}\leftarrow \alpha \sum_{i=1}^{m_{1}}y_{i}+(1-\alpha )\sum_{i=1}^{m_{2}}z_{i},$$

where we adopt the computer science notation ← to mean ‘replaces’. If *α*=0.5, then both false positives and false negatives are weighted equally. If *α*>0.5, then there is greater emphasis on correctly classifying the vectors in $M_{1}$, and the reverse if *α*<0.5. With this final problem formulation, the following desirable properties result:

— the problem is a linear programming problem and is therefore tractable even for extremely large values of *n*, the number of features;
— the formulation can be applied without knowing beforehand whether or not the dataset is linearly separable;
— the formulation provides for a trade-off between false positives and false negatives; and
— most important, the optimal weight vector *w* has at most *m* non-zero entries, where *m* is the number of samples. Hence, the classifier uses at most *m* out of the *n* features.

For these reasons, the ℓ_1_-norm SVM forms the starting point for our further research into classification.

Until now, the discussion has been on two-class classification. The SVM framework does not extend to multi-class classification very readily. For one thing, when there are only two classes, it does not matter which class is labelled ‘positive’ and which is labelled ‘negative’. On the other hand, when there are multiple classes, it is necessary to distinguish between two cases: in the first, there is a natural ordering among the class labels. For instance, if a patient's response is to be categorized as poor, fair, good, very good and excellent, the ordering is clear. In the second, there is no natural ordering, for example, if one wishes to assign a breast cancer tumour into one of the four subtypes mentioned above. The paper [51] is among the more popular methods for multi-class SVM.

### (c) Some applications of support vector machines to cancer

In contrast with sparse regression, there are many applications of sparse classification methods to cancer biology. A search of the Pubmed database of the National Library of Medicine, USA, with the string ‘SVM cancer’ returns several hundred results. The vast majority of these papers present applications where human experts pare the hundreds or even thousands of measured features to a small subset, to which a standard ℓ_2_-norm SVM is applied. In other words, though the raw number of measured features is very high, the actual number of features used by the SVM is smaller than the number of samples. In principle, the ℓ_1_-norm SVM can be applied to the original feature set to choose the most predictive features out of the overall feature set. But for the most part, existing applications appear to leave the task of feature selection to human experts and not to the algorithm. The fraction of SVM applications that exploit the feature selection property of the ℓ_1_-norm SVM is quite small. Two reasons, not mutually exclusive, can be proposed to explain this. First, the users might simply be unfamiliar with the ℓ_1_-norm SVM methodology. Second, the users might believe that a purely data-driven approach might result in a feature set whose biological significance is unclear.

The paper [48] that introduces the technique of RFE to the SVM algorithm studies the application of the technique to leukaemia. It is shown that the SVM–RFE method eventually leads to just two features being retained. However, several authors report that the performance of the SVM–RFE approach is in general somewhat erratic. It would appear therefore that the dataset studied in Guyon *et al.* [48] is particularly amenable to the use of this technique. An excellent review of several applications of SVMs to ovarian cancer can be found in Sabatier *et al.* [52]. Other examples of ovarian cancer applications include Han *et al.* [53], in which 322 samples are analysed to generate a 349-gene biomarker panel which performs very well, but when the 349 genes are reduced to 18 genes the performance on the test data is poor; Denkert *et al.* [54], in which a 300-gene ovarian carcinoma index is constructed on the basis of 80 samples, which is then tested on 118 samples; and Hartmann *et al.* [55], in which a panel of 14 genes is identified to differentiate between early relapse and late-stage relapse. Yet, these papers ignore the result from Wenocur & Dudley [44], which states that if the number of features used is in excess of the number of samples in the training data, then generically an SVM can achieve 100% accuracy, sensitivity and specificity on the training data, irrespective of the assignment of labels to the samples. As neither Han *et al.* [53] nor Denkert *et al.* [54] reports such a phenomenon, it is unclear whether these papers have implemented the SVM algorithm accurately.

As illustrations of applications to other forms of cancer, one can mention: Sabatier *et al.* [56], in which a 368-gene expression signature is trained on 2145 basal breast cancer samples, and then tested on another set of 2034 samples, with the aim of predicting the patient's prognosis; and Klement *et al.* [57], in which seven features were selected by human experts to study 399 NSCLC patients. As an example of using not just SVM but also RFE, one can cite Yang *et al.* [58], in which the efficacy of drug candidates against hepatocellular carcinoma (liver cancer) is studied. As the efficacy of drug candidates is actually a real number between 0 and 1, the authors discretize the efficacy into two bins: [0,0.4] and [0.6,1]. Apparently, their only motivation is to make the problem fit into the SVM framework. Therefore, it would be worthwhile to apply sparse regression techniques (as opposed to sparse classification) to such problems. Finally, an application of the multi-class SVM methodology of Crammer & Singer [51] is found in Huang *et al.* [59].

### (d) The lone star algorithm

As pointed out in §4, both the traditional ℓ_2_-norm SVM and the ℓ_1_-norm SVM can be used for two-class classification problems. When the number of samples *m* is far larger than the number of features *n*, the traditional SVM performs very satisfactorily, whereas the ℓ_1_-norm SVM of Bradley & Mangasarian [49] is to be preferred when *m*<*n*. Moreover, the ℓ_1_-norm SVM is guaranteed to use no more than *m* features. However, in many biological applications, even *m* features are too many. Biological measurements suffer from poor repeatability. Therefore, a classifier that uses fewer features would be far preferable to one that uses more features. In this section, we present a new algorithm for two-class classification that often uses far fewer than *m* features, thus making it very suitable for biological applications. The algorithm combines the ℓ-norm SVM of Bradley & Mangasarian [49], RFE of Guyon *et al.* [48] and stability selection of Meinshausen & Bühlmann [60]. A preliminary version of this algorithm was reported in Ahsen *et al.* [61]. Note that Li *et al.* [62] introduces an algorithm known as SVM–T–RFE that contains some similarities to this algorithm.

The algorithm is as follows:

(1) Choose at random a ‘training set’ of samples of size *k*_1_ from $M_{1}$ and size *k*_2_ from $M_{2}$, such that *k*_*l*_≤*m*_*l*_/2 and *k*_1_,*k*_2_ are roughly equal. Repeat this choice *s* times, where *s* is a ‘large’ number. This generates *s* different ‘training sets’, each of which consists of *k*_*l*_ samples from $M_{l}$, *l*=1,2.
(2) For each randomly chosen training set, compute a corresponding optimal ℓ_1_-norm SVM using the formulation (4.2). This results in *s* different optimal weight vectors and thresholds.
(3) Let *k* denote the average number of non-zero entries in the optimal weight vector across all randomized runs. Average all *s* optimal weight vectors and thresholds, retain the largest *k* components of the averaged weight vector and corresponding feature set, and set the remaining components to zero. This results in reducing the number of features from the original *n* to *k*.
(4) Repeat the process with the reduced feature set, but the originally chosen randomly selected training samples, until no further reduction is possible in the number of features. This determines the final set of features to be used.
(5) Once the final feature set is determined, carry out twofold cross validation by dividing the data *s* times into a training set of *k*_1_,*k*_2_ randomly selected samples and assessing the performance of the resulting ℓ_1_-norm classifier on the testing dataset, which is the remainder of the samples. Average the weights generated by the *t*≤*s* best-performing classifiers, where *t* is chosen by the user, and call that the final classifier.

When the number of features *n* is extremely large, an optional pre-processing step is to compute the mean value of each of the *n* features for each class, and retain only those features wherein the difference between means is statistically significant using the ‘Student’ *t*-test. Our experience is that using this optional pre-processing step does not change the final answer very much, but does decrease the CPU time substantially. Note that, in Li *et al.* [62], a weighted combination of the weight of the ℓ_2_-norm SVM and the *t*-test statistic is used to eliminate features.

Now some comments are in order regarding the above algorithm:

— in some applications, $M_{1}$ and $M_{2}$ are of comparable size, so that the size of the training set can be chosen to equal roughly half of the total samples within each class. However, in other applications, the sizes of the two sets are dissimilar, in which case the larger set has far fewer of its samples used in training;
— step 1 of randomly choosing *s* different training sets differs from Guyon *et al.* [48], where there is only one randomized division of the data into training and testing sets;
— for each random choice of the training set, the *number* of non-zero entries in the optimal weight vector is more or less the same; however, the *locations* of non-zero entries in the optimal weight vector vary from one run to another;
— in step 3 above, instead of averaging the optimal weights over all *s* runs and then retaining the *k* largest components, it is possible to adopt another strategy. Rank all *n* indices in order of the number of times that index has a non-zero weight in the *s* randomized runs, and retain the top *k* indices. In our experience, both approaches lead to virtually the same choice of the indices to be retained for the next iteration;
— instead of choosing *s* randomized training sets right at the outset, it is possible to choose *s* randomized training sets each time the number of features is reduced; and
— in the final step, there is no distinction between the training and testing datasets, so the final classifier is run on the entire dataset to arrive at the final accuracy, sensitivity and specificity figures.

The advantage of the above approach vis-à-vis the ℓ_2_-norm SVM–RFE of Guyon *et al.* [48] or the SVM–T–RFE of Li *et al.* [62] is that the number of features reduces significantly at each step, and the algorithm converges in just a few steps. This is because, in the ℓ_1_-norm SVM, many components of the weight vector are ‘naturally’ zero and need not be truncated. By contrast, in general all the components of the weight vector resulting from the ℓ_2_-norm SVM will be non-zero; as a result, the features can only be eliminated one at a time, and in general the number of iterations is equal to (or comparable to) *n*, the initial number of features.

The new algorithm can be appropriately referred to as the ‘ℓ_1_-SVM *t*-test and RFE’ algorithm, where SVM and RFE are themselves acronyms as defined above. Once again taking the first letters, we are led to the ‘second-level’ acronym ‘ℓ_1_-StaR’, which can be pronounced as ‘ell-one star’. Out of deference to our domicile, we have decided to call it the ‘lone star’ algorithm.

The lone star algorithm was applied to the problem of predicting which patients of endometrial cancer are at risk of lymph node metastasis. These results are reported elsewhere. But in brief, the situation is the following: the endometrium is the lining of the uterus. When a patient contracts endometrial cancer, her uterus, ovaries and fallopian tubes are surgically removed. One of the major risks run by endometrial cancer patients is that the cancer will metastasize and spread through the body via pelvic and/or para-aortic lymph nodes. The Gynecologic Oncology Group recommends that the patient's pelvic and para-aortic lymph nodes should also be surgically removed when the size of the tumour exceeds 2 cm in diameter. However, post-surgery analysis reveals that, even in this case, lymphatic metastasis is present in only 22% of the cases [63].

To predict the possibility of lymphatic metastasis, 1428 miRNAs were extracted from 94 tumours, half with and half without metastasis. Using the lone star algorithm, 13 miRNAs were identified as being highly predictive. When tested on the entire training sample of 94 tumours, the lone star classifier correctly classified 41 out of 43 lymph-positive samples, and 40 out of 43 lymph-negative samples. In ongoing work, these miRNAs were measured on an independent cohort of 19 lymph-negative and nine lymph-positive tumours. The classifier classified eight out of nine lymph-positive tumours correctly, and 11 out of 19 lymph-negative tumours correctly. Thus, while the specificity is not very impressive, the sensitivity is extremely good, which is precisely what one wants in such a situation. Moreover, using a two-table contingency analysis and the Barnard exact test, the likelihood of arriving at this assignment by pure chance (the so-called *p*-value) is bounded by 0.011574. In biology, any *p*-value less than 0.05 is generally considered to be significant.

## 5. Some topics for further research

Both machine learning and computational biology are vast subjects, and their intersection contains many more topics than are touched upon in this brief article. Besides, there are other topics in computational cancer biology that do not naturally belong to machine learning, for example modelling tumour growth using branching processes. Therefore, the emphasis in this article has been on topics that are well established in the machine learning community and are also relevant to problems in computational cancer biology.

Until now several ‘penalty’ norms have been proposed for inducing an optimization algorithm to select structured sparse feature sets, such as GL and SGL. As pointed out in §2, available extensions of these penalty norms to overlapping sets do not address biological networks where there are multiple paths from a master regulator to a final node. Any advance in this direction would have an immediate application to computational biology.

Compressed sensing theory as discussed in §3 is based on the premise that it *is possible to choose* the measurement matrix *A*. The available theorems in this theory are based on assumptions on the measurement matrix, such as the RIP, or the null space property, and perhaps something even more general in future. In order to apply techniques from compressed sensing theory to cancer biology, it would be necessary to modify the theory to the case where the measurement matrix is given, and not chosen by the user. The RIP corresponds to the assumption that in an *m*×*n* matrix *A*, every choice of *k* columns results in a nearly orthogonal set. In actual biological data, such an assumption has no hope of being true, because the expression levels of some genes would be highly correlated with those of other genes. In Candès & Plan [36], the authors suggest that it is possible to handle this situation by first clustering the column vectors and then choosing just one exemplar from each cluster before applying the theory. Our preliminary attempts to apply such an approach to ovarian cancer data [29] are not very promising, leading to RIP orders of 5 or 10—far too small to be of practical use. Thus, there is a need for the development of other heuristics besides clustering to extract nearly orthogonal sets of columns for actual measurement matrices. In this connection, it is worth pointing out [64] that group RIP is easier to achieve using random projections, as compared with RIP. However, it is not clear whether a ‘given’ *A* matrix is likely to satisfy a group RIP with a sufficiently large order.

In general, it would appear that sparse regression is more advanced than sparse classification, with both well-established theoretical foundations as well as widely used algorithms in the former. By contrast, sparse classification does not have such a wealth of results. The lone star algorithm introduced here has performed well in several applications involving cancer data, and, at least for the moment, it appears to be the only available method to select far fewer features than the size of the training set of samples. As of now, there is no theoretical justification for this observed behaviour. Recall that the ℓ_1_-norm SVM is guaranteed only to choose no more features than the size of the training set; but there is no reason to assume that it will use fewer. Therefore, it is certainly worthwhile to study when and why lone star and other such algorithms will prove to be effective.

## Footnotes

1 See Malhotra *et al.*[7] for a more refined partitioning into six subtypes. However, the refined subtyping involves other genes, not only these three.

2 My biology collaborator, Prof. Michael A. White of the UT Southwestern Medical Center, says that the same is true in the opposite direction as well. He and his students find it easier to understand algorithms by just talking to me and my students. Perhaps there is a lesson in that.

3 There are situations, such as transcription factor genes regulating other genes, where taking such a product would be realistic. But such situations are relatively rare.

4 Note that no penalty is imposed on the threshold *θ*.

5 In Davenport *et al.*[30], it is suggested that a precursor of compressed sensing can be found in a paper that dates back to 1795!

## Author profile

 **Mathukumalli Vidyasagar** is the Cecil and Ida Green (II) Professor of Systems Biology Science at the University of Texas at Dallas. His current research interests are in the application of stochastic processes and stochastic modelling to problems in computational biology, and control systems.

He was previously director of the newly created Centre for Artificial Intelligence and Robotics (CAIR) in Bangalore, under the Ministry of Defence, Government of India, and built up CAIR into a leading research laboratory, working in areas such as flight control, robotics, neural networks and image processing. He then moved to the Indian private sector as an Executive Vice President of India's largest software company, Tata Consultancy Services. In the city of Hyderabad, he created the Advanced Technology Center, an industrial R&D laboratory of around 80 engineers, working in areas such as computational biology, quantitative finance, e-security, identity management and open source software to support Indian languages.

Vidyasagar has received a number of awards in recognition of his research contributions, including election to the Fellowship of the Royal Society in 2012, the IEEE Control Systems (Field) Award, the Rufus Oldenburger Medal of ASME and others. He is the author of 11 books and nearly 140 papers in peer-reviewed journals.
